# EHMT1 regulates Parvalbumin-positive interneuron development and GABAergic input in sensory cortical areas

**DOI:** 10.1007/s00429-020-02149-9

**Published:** 2020-09-25

**Authors:** Moritz Negwer, Karol Piera, Rick Hesen, Lukas Lütje, Lynn Aarts, Dirk Schubert, Nael Nadif Kasri

**Affiliations:** 1 Department of Human Genetics, Radboudumc, Donders Institute for Brain, Cognition, and Behaviour, 6500 HB Nijmegen, The Netherlands; 2 Department of Cognitive Neuroscience, Radboudumc, Donders Institute for Brain, Cognition and Behaviour, 6500 HB Nijmegen, The Netherlands

**Keywords:** Parvalbumin-positive interneurons, Kleefstra syndrome, Gabaergic synapse, Critical period, Cortical development, Autism spectrum disorder, EHMT1

## Abstract

Mutations in the Euchromatic Histone Methyltransferase 1 *(EHMT1)* gene cause Kleefstra syndrome, a rare form of intellectual disability (ID) with strong autistic traits and sensory processing deficits. Proper development of inhibitory interneurons is crucial for sensory function. Here we report a timeline of Parvalbumin-positive (PV^+^) interneuron development in the three most important sensory cortical areas in the *Ehmt1*^+*/*−^ mouse. We find a hitherto unreported delay of PV^+^ neuron maturation early in sensory development, with layer- and region-specific variability later in development. The delayed PV^+^ maturation is also reflected in a delayed maturation of GABAergic transmission in *Ehmt1*^+*/*−^ auditory cortex, where we find a reduced GABA release probability specifically in putative PV^+^ synapses. Together with earlier reports of excitatory impairments in *Ehmt1*^+*/*−^ neurons, we propose a shift in excitatory-inhibitory balance towards overexcitability in *Ehmt1*^+*/*−^ sensory cortices as a consequence of early deficits in inhibitory maturation.

**Electronic supplementary material:**

The online version of this article (10.1007/s00429-020-02149-9) contains supplementary material, which is available to authorized users.

## Introduction

Kleefstra Syndrome (KS, OMIM#610253) is a rare syndromic form of intellectual disability (ID) associated with autistic features (Kleefstra et al. 2005, 2012; Koemans et al. 2017; Vermeulen et al. 2017), caused by haploinsufficiency of the *EHMT1* gene. The EHMT1 protein (also known as GLP or KMT1D) functions as a repressive epigenetic modifier by mono- and dimethylating euchromatic Histone 3 on Lysine 9 (H3K9me1/2; Benevento et al. 2015; Iwase et al. 2017; Shinkai and Tachibana 2011; Tachibana et al. 2005). Mice with a heterozygous loss-of-function mutation in the *Ehmt1* gene recapitulate the core phenotypes of KS, including low muscle tone, delayed development of sensory modalities and craniofacial abnormalities (Balemans et al. 2010, 2014). *Ehmt1*^+*/*−^ mice show a delayed onset of sensory experiences (eye opening) and sensory reaction (acoustic startle response; Balemans et al. 2014), as well as impaired learning and exploration, reduced locomotor activity, and increased anxiety (Balemans et al. 2013). This phenotype is partially replicated in excitatory-neuron specific *Ehmt1* knockout mice (CaMKIIa-Cre), which also present with hypoactivity and learning deficits, but do not show an anxiety-like phenotype (Schaefer et al. 2009).

On a physiological level, we previously reported hyperexcitability in *Ehmt1*^+*/*−^ hippocampal excitatory neurons (Frega et al. 2020) as well as deficits in evoked excitatory transmission in auditory cortex (Frega et al. 2019). Those excitatory phenotypes are broadly replicated in cultured neurons derived from both rodents and human KS patient-derived iPS cells (Frega et al. 2019; Martens et al. 2016). *Ehmt1*^+*/*−^ neurons show a deficit in homeostatic synaptic scaling (upscaling at excitatory synapses) in response to input deprivation, both in primary cultures and in the visual cortex (Benevento et al. 2016). On a molecular level, *Ehmt1* and its homolog *Ehmt2* co-regulate activity-dependent gene expression in the hippocampus and amygdala (Gupta-Agarwal et al. 2012, 2014).

Interestingly, the deficit in sensory development observed in *Ehmt1*^+*/*−^ mice (Balemans et al. 2014) was not observed in excitatory-neuron specific *Ehmt1* conditional knockout mice (*Ehmt1*^*flox/*+^ *x* *CaMKIIa-Cre*; Schaefer et al. 2009), suggesting an inhibitory component for the developmental deficit. This hypothesis is strengthened by recent physiological evidence of an inhibitory impairment in the *Ehmt1*^+*/*−^ hippocampal stratum oriens (Frega et al. 2020), which is enriched in PV^+^ basket cells (Booker and Vida 2018), indicating PV^+^ neurons as a possible target subpopulation. In sensory cortical areas, PV^+^ interneurons set the pace for the maturation of the entire circuit, which might explain the sensory developmental deficit observed in *Ehmt1*^+*/*−^ mice. However, the influence of *Ehmt1* on the trajectory of different inhibitory interneuron classes remains unknown.

Parvalbumin-positive (PV^+^) interneurons are the largest group of GABAergic interneurons (releasing γ-amino butyric acid) in the cortex and directly control firing in their target cells (Tremblay et al. 2016). They mature relatively late during cortical development, during a time of heightened sensitivity to environmental influences known as the critical period (Hensch 2005; Marín 2016; Reh et al. 2020; Trachtenberg 2015). During the critical period, PV^+^ neurons start expressing the calcium-buffering protein PV and generate perineuronal nets (PNNs), a honeycomb-like extracellular matrix structure that elevates their firing rate and effectively locks in their excitatory input at this timepoint (Favuzzi et al. 2019; Sigal et al. 2019; Testa et al. 2019). The timing of PV^+^ interneuron maturation is plastic and depends on sensory input (Marín 2016). Interestingly, mouse models for ID and autism spectrum disorders (ASD) show temporal shifts in both directions: earlier maturation in *Mecp2*^−/y^ mice, a mouse model for Rett Syndrome (Krishnan et al. 2015, 2017; Patrizi et al. 2019), and delayed maturation in *Fmr1*^*−/y*^ mice, a mouse model for Fragile X Syndrome (Goel et al. 2018; Harlow et al. 2010; Reinhard et al. 2019). Strikingly, PV^+^ neuron-specific knockout of *Mecp2* prevented a critical period altogether (Banerjee et al. 2016; He et al. 2014), underlining PV^+^ neuron development in sensory cortices as a possible point of convergence in neurodevelopmental disorders.

Here, we investigated the developmental trajectory of PV^+^ neurons in the three largest primary sensory cortical areas (A1, S1, V1), at three relevant timepoints for their respective development. In order to quantify inhibitory neurotransmission, we also measured the output of PV^+^ neurons onto pyramidal neurons in auditory cortex layer 2/3. We find that in *Ehmt1*^+*/*−^ mice, PV^+^ neuron maturation is delayed at postnatal day (P) 14 in layer 4 in all regions examined. With the exception of S1 layer 4, PV^+^ maturation markers in *Ehmt1*^+*/*−^ mice normalize to wild-type levels later in development. Genetic labelling of PV^+^ cells showed no difference in any region in adulthood, indicating a dynamic effect of *Ehmt1* on PV^+^ expression. In agreement with this delay of PV^+^ neuron maturation, we find a reduction of inhibitory synaptic function in the *Ehmt1*^+*/*−^ auditory cortex at P14. As a cause, we identify a reduced release probability for GABAergic synapses, specifically from fast-spiking (putative PV^+^) neurons.

## Materials and methods

### Animals

For the animal experiments presented in this study, mice heterozygous for a targeted loss-of-function mutation in the *Ehmt1* gene (*Ehmt1*^+*/*−^ mice; Tachibana et al. 2005) and their wild-type littermates (*Ehmt1*^+*/*+^) on a C57BL/6 background were used, as previously described (Balemans et al. 2010; Benevento et al. 2016). We used mice from both sexes for our study. Animals were kept in wire-top cages (type III), group-housed with either the mother animals with her nest until weaning (P21–25), and afterwards group-housed (3–6 animals, littermates, both genotypes, segregated by sex) in wire-top type III cages. Animals had access to rodent chow and water ad libitum and were kept on a 12 h/12 h light-/dark cycle (lights on at 07:00 a.m.). Animal experiments were conducted in conformity with the Animal Care Committee of the Radboud University Nijmegen Medical Centre and the National Committee on Animal Experiments (CCD), The Netherlands, and conform to the guidelines of the Dutch Council for Animal Care and the European Communities Council Directive 2010/63/EU.

### Immunostaining

*Ehmt1*^+*/*+^ and ^+*/*−^ littermates of both sexes were harvested in one of three age groups: P14, P28, and P56. Animals were deeply anesthetized with isoflurane, then decapitated and the brain was quickly extracted. The brains were fixed overnight in 4% PFA with 4% Sucrose in PBS at 4 °C and kept in PBS at 4 °C until slicing. For slicing, the brains were embedded in 1.5% Agarose in PBS, and 60 µm coronal sections were cut on a vibratome (Leica VT1000s). The slices were matched to the Allen Adult Mouse Brain Atlas (Atlas version 2, Oh et al. 2014; https://atlas.brain-map.org/atlas?atlas=1) and kept in PBS with 0.001% Sodium Azide at 4 °C.

For expression of PV and PNNs, 60 µm free-floating slices were stained overnight at room temperature with Rabbit anti-PV (Swant 27, 1:500) and biotinylated *Wisteria* *Floribunda* Agglutinin (WFA, Sigma L1516, 1:500), followed by Donkey anti-Rabbit Alexa 488 (Invitrogen A21206, 1:500), and Streptavidin-Alexa 568 (Molecular Probes S11226, 1:500) for 3 h at room temperature, and counterstained with Hoechst 33,342 (1:5000, 15 min).

For synapse staining P14 slices containing primary auditory cortex were stained as described earlier (van Rhijn et al. 2018), including an antigen retrieval step with 0.05% Trypsin in PBS (37 °C for 15 min). Slices were then incubated for 72 h at 4 °C with Chicken Anti-NeuN (Millipore ABN91, 1:750) and Mouse Anti-Gad67 (Merck Millipore MAB 5406, 1:500), followed by 3 h at room temperature with Goat anti-chicken 568 (Invitrogen A11041, 1:500), and Goat anti-mouse 647 (Invitrogen A21236, 1:500). Nuclei were counterstained with Hoechst 33,342 (1:5000, 15 min).

### Fluorescence microscopy

The PV/PNN stained slices were imaged at 5 × and 40 × on a Zeiss AxioImager.Z1 with Apotome (40 × only). We used Zeiss EC Plan-Neofluar 5 ×/0.16 M27 and Zeiss EC Plan-Neofluar 40x/0.75 M27 objectives, and imaged with a Zeiss Axiocam 506. From each sensory cortex, we collected between 1 and 10 non-overlapping images (number matched within each region to account for differences in the size of the brain region) for both layer 2/3 and 4, from each slice. Per animal, we used 4 slices per area, and per timepoint *N* = 3–6 each *Ehmt1*^+*/*+^/*Ehmt1*^+*/*−^ mice, for a total of between 13 and 68 images (matched within one brain region) per genotype for each area and layer.

The NeuN/Gad65 stained slices were imaged at 5 × and 63 × on a Zeiss AxioImager.Z1 with Apotome unit (63 × only). We used Zeiss EC Plan-Neofluar 5x/0.16 M27 and Zeiss Plan-Apochromat 63 ×/1.4 Oil DIC M27 objectives, and imaged with a Zeiss Axiocam 506. We used one timepoint (P14) with *N* = 3/3 *Ehmt1*^+*/*+^/*Ehmt1*^+*/*−^ mice, 4 slices per animal, and collected between 2 and 3 non-overlapping images of each auditory cortex layer 2/3 at 63×.

### Image analysis

**Fig. 1:**
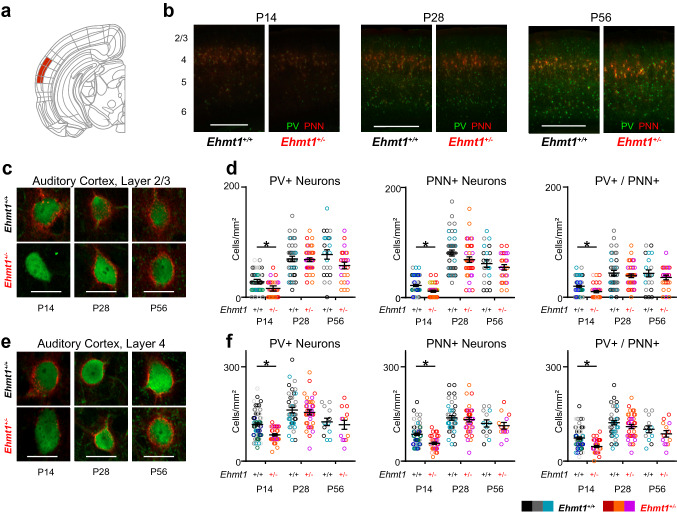
*Ehmt1* haploinsufficiency delays PV and PNN expression in the auditory cortex during the auditory critical period. **a** Primary auditory cortex, layers 2/3 and 4 are marked in red. **b** Overview of PV^+^ (green) and PNN^+^ (red) development in the auditory cortex over time. Layer numbers are marked on the left. **c** Neurons stained for Parvalbumin (green) and PNNs (red) in the primary auditory cortex, layer 2/3. **d** Quantification of the density of PV^+^, PNN^+^, and PV/PNN double-labelled cells. N P14 = 63/38, P28 = 40/39, P56 = 22/24 images, from 1 to 4 slices per animal, P14 = 6/4, P28 = 3/3, P56 = 3/3 *Ehmt1*^+*/*+^*/ Ehmt1*^+*/*−^ animals. **e** Neurons stained for Parvalbumin (green) and PNNs (red) in the primary auditory cortex, layer 4. **f** Quantification of the density of PV^+^, PNN^+^, and PV/PNN double-labelled cells. N P14 = 66/38, P28 = 40/37, P56 = 13/13 images, from 1 to 4 slices per animal, P14 = 6/4, P28 = 3/3, P56 = 3/3 *Ehmt1*^+*/*+^*/ Ehmt1*^+*/*−^ animals. Source for **a** Allen Adult Mouse Brain Atlas, https://atlas.brain-map.org/atlas?atlas=1. Image Credit: Allen Institute. Scale bars in **b** 500 µm, **c**, **e** 20 µm. Note that the intensities in **c**, **e** are matched within a timepoint, but not across timepoints. Scale in **d**, **f** cells/mm^2^, Dot plot with mean ± SEM, color-coded per mouse. *p < 0.05, **p < 0.01; Nested ANOVA with mice as subgroups. See Supplementary Table 1 for exact values

**Fig. 2:**
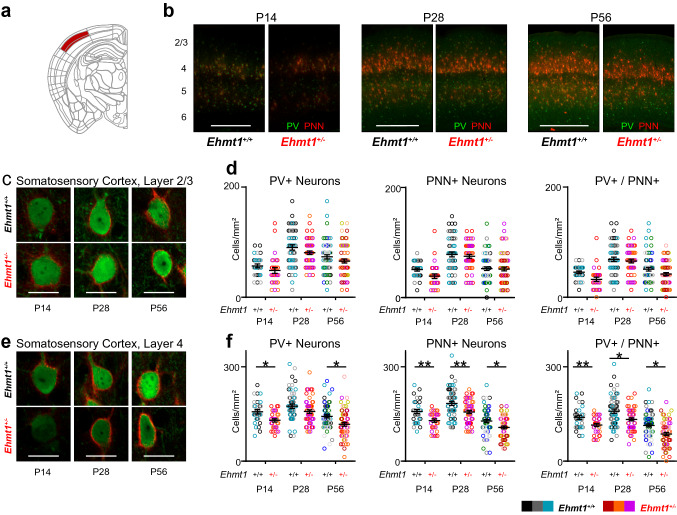
A persistent reduction in PV^+^ neuron markers in *Ehmt1*^+*/*−^ somatosensory cortex layer 4, but not in layer 2/3. **a** Primary somatosensory cortex, Barrel field, layers 2/3 and 4 are marked in red. **b** Overview of PV^+^ (green) and PNN^+^ (red) development in the somatosensory cortex over time. Layer numbers are marked on the left. **c** Neurons stained for Parvalbumin (green) and PNNs (red) in the primary somatosensory cortex, layer 2/3. **d** Quantification of the density of PV^+^, PNN^+^, and PV/PNN double-labelled cells. N P14 = 30/28, P28 = 54/53, P56 = 63/63 images, four slices each from P14 = 3/3, P28 = 3/3, P56 = 6/6 *Ehmt1*^+*/*+^*/ Ehmt1*^+*/*−^ animals. Color-code per mouse. **e:** Neurons stained for Parvalbumin (green) and PNNs (red) in the primary somatosensory cortex, layer 4. **f** Quantification of the density of PV^+^, PNN^+^, and PV/PNN double-labelled cells. N P14 = 29/29, P28 = 53/52, P56 = 68/63 images, four slices each from P14 = 3/3, P28 = 3/3, P56 = 6/6 *Ehmt1*^+*/*+^*/ Ehmt1*^+*/*−^ animals. Color-code per mouse. Source for **a** Allen Adult Mouse Brain Atlas, https://atlas.brain-map.org/atlas?atlas=1. Image Credit: Allen Institute. Scale bars in **b** 500 µm, **c**, **e** 20 µm. Note that the intensities in **c**, **e** are matched within a timepoint, but not across timepoints. Scale in **d**, **f** cells/mm^2^, Dot plot with mean ± SEM, color-coded per mouse. *p < 0.05, **p < 0.01; nested ANOVA with mice as subgroups. See Supplementary Table 1 for exact values

**Fig. 3:**
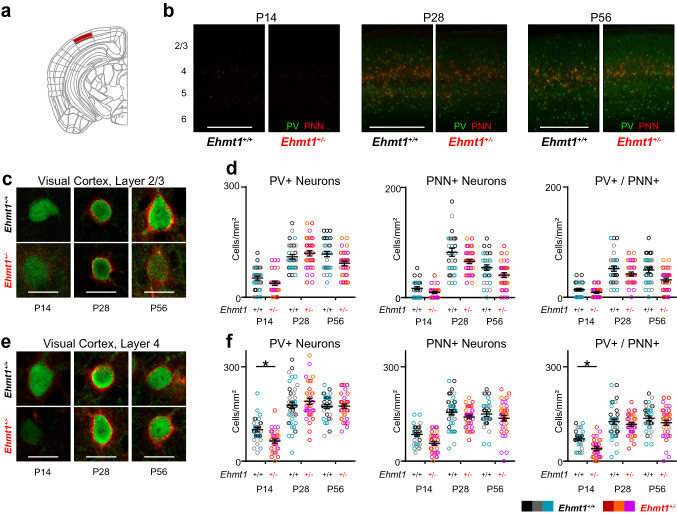
An early delay in PV + maturation in *Ehmt1*^+*/*−^ visual cortex layer 4, but not layer 2/3. **a** Primary visual cortex, layers 2/3 and 4 are marked in red. **b** Overview of PV^+^ (green) and PNN^+^ (red) development in the visual cortex over time. Layer numbers are marked on the left. **c** Neurons stained for Parvalbumin (green) and PNNs (red) in the primary Visual cortex, layer 2/3. **d** Quantification of the density of PV^+^, PNN^+^, and PV/PNN double-labelled cells. N P14 = 30/28, P28 = 32/35, P56 = 30/32 images, 12/12 slices from 3/3 *Ehmt1*^+*/*+^*/ Ehmt1*^+*/*−^ animals per age. **e** Neurons stained for Parvalbumin (green) and PNNs (red) in the primary Visual cortex, layer 4. **e**, **f** Quantification of the density of PV^+^, PNN^+^, and PV/PNN double-labelled cells. N P14 = 30/29, P28 = 39/35, P56 = 31/34 images, 12/12 slices from 3/3 *Ehmt1*^+*/*+^*/ Ehmt1*^+*/*−^ animals per age. Source for **a** Allen Adult Mouse Brain Atlas, https://atlas.brain-map.org/atlas?atlas=1. Image Credit: Allen Institute. Scale bars in **b** 500 µm, **c**, **e** 20 µm. Note that the intensities in **c**, **e** are matched within a timepoint, but not across timepoints. Scale in **d**, **f** cells/mm^2^, Dot plot with mean ± SEM, color-coded per mouse. *p < 0.05, **p < 0.01; Nested ANOVA with mice as subgroups. Please see Supplementary Table 1 for exact values

For the PV/PNN stainings, we processed the images with Zen (Blue edition, version 2.3, Zeiss) to export TIFF images. We used a custom-made ImageJ/FIJI script to randomize the file names, for blinding to the genotype. The images were analyzed semi-automatically with the PIPSQUEAK toolbox which is specifically written for PV/PNN overlap analysis (Slaker et al. 2016). Within PIPSQUEAK, we assigned the positions of PV^+^ and PNN^+^ neurons by hand without thresholding, and used PIPSQUEAK to assess PV/PNN overlap. PIPSQUEAK assignment was done blinded to the genotype. Un-blinding and further processing of the data was done in Excel, and statistics were done in Excel and Graphpad Prism (version 7). The overview images used in Figs. 1, 2 and 3b were stitched from 5 × Images with a custom-written ImageJ macro using the ImageJ stitching plugin (Preibisch et al. 2009). For the NeuN/Gad65 stainings, we processed the images with Zen (Blue edition, version 2.3, Zeiss) to export TIFF images and counted perisomatic Gad67^+^ puncta with the CellCounter plugin of ImageJ/FIJI (Schindelin et al. 2012), and the resulting counts were processed in Excel.

### Whole-brain immunostaining, clearing and analysis

For this protocol we crossed mice expressing the *tdTomato*^*flox/flox*^ conditional marker (B6.Cg-Gt(ROSA)26Sortm9(CAG-tdTomato)Hze/J or Ai9; a kind gift from Michael Valente, RIMLS Nijmegen) with *Ehmt1*^+*/*−^ mice until we had *Ehmt1*^+*/*−^; *tdTomato*^*flox/flox*^ males (Supplementary Fig. S2). Those were then crossed with *PV-Cre* homozygote females (a kind gift from Tansu Celikel, Donders Institute Nijmegen) and the resulting litter-matched offspring (*PV*^*Cre/−*^*;* *tdTomato*^*flox/−*^ and either *Ehmt1*^+*/*+^ or *Ehmt1*^±^) were used for experiments. Those mice express tdTomato in cells once the *PV* promoter has been active, and importantly, will keep expressing it even if *PV* is downregulated again, allowing us to genetically target the superset of all potential PV^+^ neurons in the cortex. At P56, the mice were sacrificed and brains were extracted and fixed as described above. We processed one hemisphere per brain following the iDISCO+ protocol (Renier et al. 2016) as described for adult brains, with all buffers according to the protocol and all incubation steps taking place on a shaker/rotor, and lasting 1 h unless mentioned otherwise. Briefly, the whole hemispheres dehydrated in a Methanol gradient, delipidated with 66% Dichloromethane (DCM, Sigma)/33% Methanol (Sigma) overnight, bleached in 5% H_2_O_2_ in Methanol at 4C overnight, then rehydrated. Hemispheres were subsequently permeabilized for 5 days at RT, blocked for 4 days at 37 °C, then incubated with primary antibodies (Rabbit Anti-RFP, Rockland 600-401-379, 1:2000, 2 ml/sample) for 6 days at 37 °C. Subsequently, brains were washed 5 × 1 h + 1 × overnight at RT, and incubated for 7 days with secondaries (Goat anti-rabbit Alexa-568, Invitrogen A11036, 1:500, 2 ml/sample) at 37 °C. Following 5 × 1 h + 1 × overnight washing at RT, samples were dehydrated in a Methanol gradient, then twice more in 100% Methanol, 66% DCM/33% Methanol, 2 × 15 min 100% DCM, and finally cleared in 100% dibenzyl ether (DBE, Sigma) in airtight glass vials. Brains were typically transparent within 2 h, and completely cleared overnight. Alexa-568 fluorescence remained undiminished until > 12 months after clearing.

The cleared samples were imaged on a LaVision Ultramicroscope II Light-sheet microscope outfitted with a NTK Photonics white-light laser and filter sets for 488 nm and 568 nm, imaged through a long-working distance objective (LaVision) at 1.1 × magnification (effective 2.2x, NA 0.1), and recorded with an Andor Neo 5.5 cooled sCMOS camera. We imaged with a 480 nm signal for autofluorescence for alignment with the Allen Brain Atlas, and 560 nm to record the PV-TdTomato signal. We used a single light-sheet from one side at 0.54 NA, scanning at 2.95/2.95/3 µm *x*/*y*/*z* resolution (3 µm *z*-steps) with the “horizontal focus” method and 17–18 horizontal focus steps. The sample was imaged submerged in DBE in sagittal configuration, and the entire cortex fit inside a single field of view (*x*/*y*), with a typical brain producing ~ 1600 *z*-planes of 3 µm each. The resulting image stack was de-striped in ImageJ, and fed into a heavily customized version of ClearMap 1 (Renier et al. 2016), with the cells being identified in Arivis Vision4D (Arivis GmbH, https://www.arivis.com/) using the “Machine Learning Segmenter” plugin, and cell locations being re-imported to ClearMap for alignment to the atlas and cell counts per region. We analyzed specifically the primary Auditory, Somatosensory, and Visual cortices, as displayed in Supplementary Figure S2. Visualizations were created in Arivis Vision4D and ImageJ, graphs and statistics were done with Excel.

### Acute slice electrophysiology

Acute slice electrophysiology was performed as described earlier (Frega et al. 2019). We used litter-matched *Ehmt1*^+*/*+^ and *Ehmt1*^+*/*−^ mice of adolescent age (postnatal day 14–16). In brief, animals were deeply anesthetized with isoflurane, then quickly decapitated. 350-µm-thick coronal slices were cut using a microtome (HM650V, Thermo Scientific) in ice-cold “cutting and storage” ACSF containing 87 mM NaCl, 11 mM Glucose, 75 mM Sucrose, 2.5 mM KCl, 1.25 mM NaH_2_PO_4_, 0.5 mM CaCl_2_, 7 mM MgCl_2_, and 26 mM NaHCO_3_, continuously oxygenated with 95% O_2_/5% CO_2_. Slices were incubated for 1 h at 32 °C, after which they were allowed to cool down to room temperature. The slices were then transferred to an upright microscope (Olympus) fitted with 2.5 × and 40 × water-immersion objectives and enhanced infrared illumination (DGC, Luigs & Neumann), and incubated in “recording” ACSF (124 mM NaCl, 10 mM Glucose, 3 mM KCl, 1.25 mM NaH_2_PO_4_, 2 mM CaCl_2_, 1 mM MgCl_2_, and 26 mM NaHCO_3_) at 30 °C with added 100 µM D-AP5 (Tocris 0106) and 5.2 µM CNQX (Tocris 1045) to block NMDA and AMPA receptors, respectively.

For measuring miniature IPSCs (mIPSCs), slices were incubated in recording ACSF with 1 µM TTX (Tocris 1069) to block action potentials, and 100 µM D-AP5 (Tocris 0106) and 5.2 µM CNQX (Tocris 1045) to block excitatory transmission. Slices were allowed to adjust to the conditions for at least 10 min prior to patching. Pyramidal cells in auditory cortex layer 2/3 were identified based on their perisomatic appearance at 40 × magnification, and patched using 3–6 MΩ borosilicate pipettes filled with a Cesium-based intracellular solution containing 115 mM CsMeSO_3_, 20 mM CsCl_2_, 10 mM HEPES, 2.5 mM MgCl_2_, 4 mM Na2ATP, 0.4 mM NaGTP, 10 mM Na-Phosphocreatine, 0.6 mM EGTA, and 5 mM QX-314 (Tocris 1014). Cells were patched at a minimum depth of 30 µm from the slice surface to minimize cutting artefacts. The cells were held in voltage-Clamp mode controlled by an SEC 05-LX amplifier (NPI electronics, Tamm, Germany), low-pass filtered at 3 kHz and sampled at 20 kHz with a Power-1401 acquisition board and Signal software (CED, Cambridge, UK). *V*_h_ was adjusted to + 10 mV, and miniature inhibitory postsynaptic currents were recorded over 10 min per cell. Subsequently, the traces were exported from Signal as text files, saved as.abf with Clampfit and then loaded into MiniAnalysis. mIPSCs were identified manually, with an amplitude threshold of 8 pA, at least 300 events per cell.

For measuring evoked GABA release (PPRs), slices were incubated in recording ACSF with 100 µM D-AP5 (Tocris 0106) and 5.2 µM CNQX (Tocris 1045) added to block AMPA and NMDA-mediated currents, respectively. A tungsten bipolar stimulation electrode (CE2C55, FHC) coupled to an SD9 stimulator (Grass Instruments, RI, USA) or an ISO-STIM 01 M (NPI electronics, Tamm, Germany) was inserted into layer 2/3 of the auditory cortex, and pyramidal cells were patched in layer 2/3 next to the bipolar electrode (< 200 µm lateral distance) as described above. GABAergic currents were measured at a holding voltage of − 60 mV, and were completely abolished by application of Picrotoxin (data not shown). Stimulus duration (1–2 ms) and voltage were adjusted for a postsynaptic amplitude of ~ 200 pA. For the paired-pulse ratio measurements, we measured a stimulus pair with 50, 100, 150, 200, and 500 ms inter-stimulus interval (ISI), 5 sweeps per ISI, with 30 s pause between sweeps. We calculated the PPR as the ratio between peak amplitudes. For baseline 1, we used the average of the 500 ms before the first stimulus, and for baseline 2 we used the value directly before the second stimulus.

For the Agatoxin experiments, we patched pyramidal cells as described above, measured one PPR protocol at baseline (5 sweeps each of 5 ISIs, as above). Subsequently, we added ω-Agatoxin-IVA (Sigma A6719) to a final concentration of 400 nM (Zaitsev et al. 2007). GABAergic responses declined by ca. 85% over the following 10 min (Fig. S3), which was only the case with Agatoxin and not with control ACSF (data not shown). After 10 min incubation, we repeated the PPR protocol as described above.

**Fig. 4:**
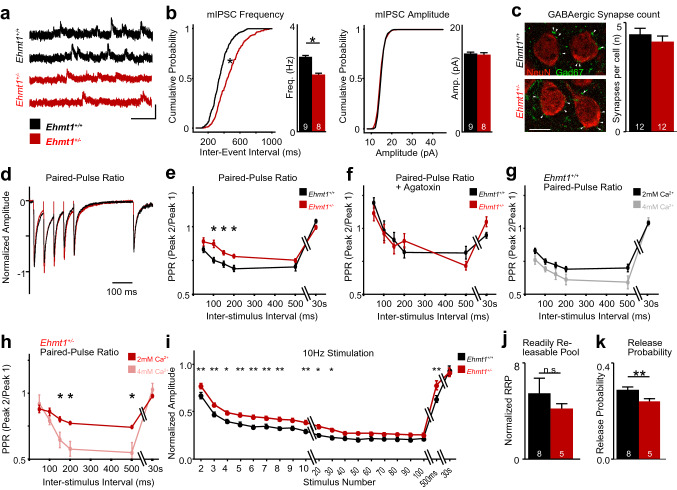
Inhibitory GABAergic transmission is impaired in *Ehmt1*^+*/*−^ auditory cortex at P14-16. **a** Representative traces of inhibitory miniature postsynaptic currents (mIPSCs) recorded from pyramidal neurons in *Ehmt1*^+*/*+^ (black) and *Ehmt1*^+*/*−^ (red). Scale bar = 20 pA, 100 ms. **b** Cumulative probability plots of mIPSC Frequency (left), and mIPSC amplitude (right). *N* = 9/8 cells. **c** Left, Putative inhibitory presynapses (arrowheads) stained with Gad67 (green) next to neuronal somata (NeuN, red). Right, quantification of perisomatic Gad67^+^ puncta. *N* = 319/237 cells in 12/12 slices from 3/3 *Ehmt1*^+*/*+^*/ Ehmt1*^+*/*−^ mice. Student’s *t* test. **d** Composite traces illustrating the paired-pulse paradigm. GABAergic inputs to A1 L2/3 pyramidal cells are stimulated at intervals of respectively 50, 100, 150, 200, or 500 ms. Scale bar = 100 ms. **e** Paired-pulse ratio for *Ehmt1*^+*/*+^ (black) and *Ehmt1*^+*/*−^ (red). *N* = 14/14 cells from 4/4 *Ehmt1*^+*/*+^*/ Ehmt1*^+*/*−^ animals. **f** Paired-pulse ratio in the presence of ω-Agatoxin-IVA, a specific blocker of P/Q type Ca^2+^ channels. **g** Comparison of *Ehmt1*^+*/*+^ PPR at physiological (2 mM, black) and elevated (4 mM, grey) extracellular Ca^2+^. **h** Comparison of *Ehmt1*^+*/*−^ PPR at physiological (2 mM, red) and elevated (4 mM, salmon) extracellular Ca^2+^. **i** Run-down of vesicle pools by 10 Hz stimulation at 4 mM extracellular Ca^2+^. Normalized amplitudes plotted against time; 100 stimuli with 100 ms ISI, followed by control pulses at 500 ms and 30 s (sweep end). Multiple *t* tests with Benjamini–Hochberg correction. **j** Readily releasable pool determined from the 10 Hz stimulus train. RRP displayed normalized to the initial stimulus amplitude. **k** Release Probability determined from the 10 Hz stimulus train. *p < 0.05, **p < 0.01. Please see Supplementary Table 2 for exact values

For the high-Ca^2+^ incubations in Fig. 4g–j, we added an additional 2 mM Ca^2+^ to the recording ACSF as described above, for a total ACSF Ca^2+^ concentration of 4 mM. The rundown experiments in Fig. 4i-k used 1-2 ms stimulus duration with initial peak amplitude adjusted to ~ 200 pA. We used a train of 100 stimuli with 100 ms ISI (10 Hz), with a recovery stimulus at 500 ms after the end of the stimulus train and 30 s inter-sweep interval, for 10 sweeps in total. The trains were then exported from Signal as txt files, and amplitudes were quantified using a custom-written Python script. All further data processing was done in Excel. For the graph shown in Fig. 4i, we normalized all amplitudes to the first peak amplitude.

We calculated the readily releasable pool and the release probability by curve-fitting on a plot of the individual response amplitude against cumulative amplitude (Elmqvist and Quastel 1965; see for review Neher 2015). To get the RRP, normalized amplitudes (*y*-axis) were plotted against normalized cumulative amplitudes (*x*-axis). The steep initial change was extrapolated linearly, and the *x*-axis intercept gives the estimate of the readily releasable pool (RRP). The slope gives the estimate of the compound release probability (*P*_R_).

### Statistics

Data were processed in Excel and Python, and statistics and graphics were made in Excel, Python (Seaborn v0.10) and Graphpad Prism (version 7). Two-sample Kolmogorov–Smirnov tests for the mIPSC cumulative probability plots in Fig. 4b were done in RStudio. Unless otherwise indicated, the graphs show mean ± SEM, and were tested for differences using either a nested ANOVA using mice as subgroups and genotypes as groups, or two-sided Student’s *t* test with Bonferroni correction for multiple testing.

## Results

We first investigated whether a heterozygous loss of *Ehmt1* influences the developmental trajectory of PV^+^ GABAergic interneurons in the sensory cortices in mice. We compared heterozygous *Ehmt1* mutant (*Ehmt1*^+*/*−^) mice to wild-type (*Ehmt1*^+*/*+^) littermates, a construct valid model for KS (Balemans et al. 2010), and because a full knockout is lethal at the embryonic stage (Tachibana et al. 2005). We selected three well-characterized sensory regions: primary auditory cortex (A1), the barrel subfield in the primary somatosensory cortex (S1), and the primary visual cortex (V1). In each region, we generated a timeline of PV^+^ neuron maturation, measuring two maturation markers, Parvalbumin (PV) and Perineuronal Nets (PNNs) in cortical layers 2/3 and 4. Both layers are crucial for sensory cortex function. In the simplest version of the canonical sensory cortical model, layer 4 is the thalamo-recipient “input” layer that first receives sensory input during development. Layer 4 neurons then send the processed input to neurons in layer 2/3, which integrate both locally and wide-range inputs. Consequentially, layer 2/3 is thought to mature later than layer 4 (Butt et al. 2017). We selected three timepoints based on the developmental trajectory of the primary auditory cortex (A1): P14 is expected to be the onset of the auditory critical period for thalamocortical input to layer 4 (Barkat et al. 2011). It is also the timepoint at which *Ehmt1*^+*/*+^ mice show an acoustic startle response (Balemans et al. 2014). The second timepoint, P28, is thought to be after the end of the auditory cortical critical period (Oswald and Reyes 2011; Kalish et al. 2020). For the third timepoint we selected P56, when mice are considered adult for the purposes of sensory development.

### Expression of PV and PNNs is delayed In *Ehmt1*^+/−^ primary auditory cortex

We first measured the developmental trajectory of PV^+^ neurons in the primary auditory cortex, in both layers 2/3 and 4 (Fig. 1a). We visualized PV by immunostaining and PNN by staining with *Wisteria* *Floribunda* Agglutinin (WFA), and counted the presence of PV^+^, PNN^+^ and PV/PNN double-labelled cells in both layers 2/3 and 4. Consistent with an ongoing developmental process, in *Ehmt1*^+*/*+^ the density of both PV^+^ and PNN^+^ cells increased from P14 to P28 and levelled off afterwards, and was higher in layer 4 compared to layer 2/3 (Fig. 1b). We found a significant reduction in the density of PV^+^ and PNN^+^ neurons at P14, both in layer 2/3 (Fig. 1 c-d) and layer 4 (Fig. 1e, f, Supplementary Fig. S1, Supplementary Table S1) in *Ehmt1*^+*/*−^ compared to *Ehmt1*^+*/*+^ mice. Consequentially, we also found a reduction in PV/PNN double-labelled cells in both layers at P14. After P14, all measures normalized to *Ehmt1*^+*/*+^ levels in both layer 2/3 (Fig. 1c, d) and layer 4 (Fig. 1e, f). The density of PV^+^ neurons in layer 4 was at a slightly lower level at P56 compared to P28 (Fig. 1f), which might indicate a dynamic downregulation as observed between P30–40 (Bhumika et al. 2020). This downregulation in layer 4 appears to be unchanged between genotypes, as we did find the same distribution at P56 both with slice immunostainings (Fig. 1f) and genetic labelling (PV^Cre^-Tdtomato^flox^, Supplementary Fig. S2c). These findings indicate that initial PV^+^ neuron maturation is delayed in *Ehmt1*^+*/*−^ auditory cortex. Interestingly, P14 marks the beginning of the formation of the tonotopic representation in the auditory cortex (Oswald and Reyes 2011), and thus the beginning of the auditory critical period (Barkat et al. 2011; Kalish et al. 2020). In agreement with a delayed auditory cortical development, *Ehmt1*^+*/*−^ mice also display a delayed acoustic startle response around this time (*Ehmt1*^+*/*+^ P13.5, *Ehmt1*^+*/*−^ P14.5; Balemans et al. 2014).

### The *Ehmt1*^+*/*−^ somatosensory cortex shows a consistent reduction of PV^+^ cell markers in layer 4, but not in layer 2/3

The somatosensory cortex is the earliest sensory cortex to mature, with input to layer 4 maturing by between P5 and P8, and layer 2/3 between P10 and P15, and mature PV^+^ function by P12 (Butt et al. 2017). The timepoints map onto late critical period for layer 2/3 (P14), post-critical period (P28) and adulthood (P56). As in the auditory cortex, we observed the largest density of PV/PNN labelled neurons in layer 4, but in contrast to auditory cortex, we also observed a double-labelled population in layer 5b (Fig. 2b). When quantifying the PV/PNN-labelled neurons in layer 2/3, we found no change at any timepoint (Fig. 2c, d, Supp. Table S1), except a lower percentage of PV^+^ neurons with PNNs at P14 (Supplementary Fig. S1). This matches with P14 being in the middle of the critical period for input to layer 2/3 (Butt et al. 2017).

In the *Ehmt1*^+*/*−^ layer 4 however, we found that all three measures were significantly reduced compared to *Ehmt1*^+*/*+^. Interestingly, this reduction in *Ehmt1*^+*/*−^ persisted throughout P28 and into adulthood at P56 (Fig. 2e, f, Supplementary Table S1), with the exception of PV^+^ at P28. We furthermore found an especially large reduction in PV^+^ and PV/PNN double-labelled neuron density in *Ehmt1*^+*/*−^ at P56 (Fig. 2f), indicative of an ongoing reduced PV^+^ expression, which does not happen in wild-type mice, even at advanced ages (Ueno et al. 2019). Thus, in contrast to all other areas studied, in the *Ehmt1*^+*/*−^ somatosensory cortex layer 4 we find a persistent PV^+^ neuron maturation deficit. This could be either due to a reduced density of PV^+^ precursor neurons (i.e. generation or migration of MGE-derived neurons), or due to a dynamic downregulation of PV^+^ expression. In order to differentiate between both options, we genetically labelled neurons in which the PV^+^ promoter had been active at any time (PV^Cre^-Tdtomato^flox^, Supplementary Figure S2a). Following whole-brain immunostaining and clearing with the iDISCO + protocol (Renier et al. 2014, 2016), we found that the PV^+^ neuron distribution was similar between *Ehmt1*^+*/*−^ and *Ehmt1*^+*/*+^ at P56 in S1, A1, or V1 (Supplementary Figure S2c). This indicates a continuous downregulation of PV in PV^+^ neurons in the *Ehmt1*^+*/*−^ S1, possibly already at our earliest timepoint P14, which is after the critical period for somatosensory cortex layer 4 (Butt et al. 2017). As a consequence, the E/I balance in the *Ehmt1*^+*/*−^ somatosensory cortex could be shifted towards over-excitability, which would predict a deficit in whisker-based sensation. This prediction is consistent with previous behavioral studies that indicate a reduction in exploration behavior and neophobia (Balemans et al. 2010, 2014).

### PV^+^ maturation is reduced early in *Ehmt1*^+*/*−^ Visual cortex layer 4

The visual cortex starts receiving sensory input after eye opening around P13, and therefore is the last primary sensory cortex to mature (Espinosa and Stryker 2012). Its critical period is typically defined as the refinement of binocular representations, which is thought to start after P21 and continue until at least P35, with some plasticity remaining long afterwards (Espinosa and Stryker 2012; Hensch 2005).

From all tested areas, the visual cortex showed the lowest overall count of neurons in both layer 2/3 and 4 at P14 (Fig. 3b), consistent with a time period in which incoming sensory input is only starting to trigger PV expression. When we quantified the density of PV/PNN labelled neurons in layer 4, we found a significant reduction of PV^+^ and PV/PNN double-labelled neuron density at P14, comparable to layer 4 in A1 and S1 (Fig. 3e, f). Similar to A1 layer 4, both PV^+^ and PV/PNN double-labelled densities caught up to *Ehmt1*^+/+^ levels by P28 (Fig. 3e, f), and at adulthood both genotypes were indistinguishable both in slice stainings (Fig. 3e, f) and genetically labelled PV^+^ neurons (Supplementary Fig. S2c).

The phenotype we found in V1 layer 4 is thus comparable to the ones found in the other sensory regions at P14. In the case of the visual cortex, a difference in the arrival of sensory input is readily apparent, where *Ehmt1*^+/−^ mice open their eyes on average at P16, approximately 2 days later than *Ehmt1*^+/+^ mice (Balemans et al. 2014). Consequentially, the *Ehmt1*^+/−^ mice measured at P14 would on average still have had their eyes closed, and therefore received less visual input than their WT littermates. Interestingly, our findings indicate that *Ehmt1*^+/−^ visual cortex PV^+^ neurons were maturing similarly to WT neurons by the onset of the visual cortex critical period (P21–35).

We then wondered whether the density reduction we observed in *Ehmt1*^+*/*−^ mice was similar across mice, or whether each *Ehmt1*^+*/*−^ mouse shows a reduction only in a subset of areas. In order to assess inter-individual variability, we normalized the densities of each *Ehmt1*^+*/*−^ mouse to the average of the *Ehmt1*^+*/*+^ mice. We plotted the PV^+^/PNN^+^ results per *Ehmt1*^+*/*−^ mouse for which we had data from all areas (Supplementary Fig. S3, Supplementary Table S1). Our findings largely mirrored the findings in Figs. 1, 2 and 3. Specifically, a general trend in the *Ehmt1*^+*/*−^ towards lower values than *Ehmt1*^+*/*+^ across all timepoints, with the most pronounced difference at P14 across all areas, and more subtle differences afterwards. Accordingly, when comparing the variation between genotypes, we observed a trend towards higher Coefficient of Variation in *Ehmt1*^+*/*−^ at P14, but not at P28 and P56 (Supplementary Table S1). Interestingly, those differences in CV at P14 appear to be driven mainly by layers 2/3 rather than layers 4. However, the normalized densities per mouse (Supplementary Fig. S3) suggest that the reductions in *Ehmt1*^+*/*−^ appear to be similar across all measured areas per mouse, rather than each mouse exhibiting reductions in a subset of areas.

### Reduced inhibitory synaptic transmission in *Ehmt1*^+*/*−^ is caused by reduced GABA release from putative PV^+^ neurons

Next, we wondered whether the delay in PV^+^ neuron maturation at P14 would lead to reduced GABAergic transmission onto pyramidal neurons. To this end, we measured miniature inhibitory postsynaptic currents (mIPSCs, Fig. 4a, b) in acute slices of the auditory cortex layer 2/3 at P14–16. Inhibitory inputs are likely to be dominated by inhibitory inputs from basket cells, most of which are fast-spiking PV^+^ neurons (Tremblay et al. 2016). However, as we have shown in Fig. 1, PV expression is still being established at P14 in A1 layer 2/3, so even though basket cells are present and provide GABAergic input, only a subset of the basket cell population will already express PV.

We found a significant reduction in mIPSC frequency in *Ehmt1*^+*/*−^ neurons, but no change in amplitude (Fig. 4a, b, Supp. Table S2). Interestingly, the reduced mIPSC frequency was not apparent when recorded at P21, after the closure of the auditory critical period in normally developing mice (Supplementary Figure S5). The reduced mIPSC frequency could be explained either by a reduced number of synapses or a reduced release probability. To distinguish between both possibilities, we first stained for perisomatic GABAergic presynapses on pyramidal cells, which mostly originate from basket-cells (present or future PV^+^). We found no difference in the number of GABAergic (Gad67^+^) puncta between genotypes (Fig. 4c), suggesting that the change in mIPSC frequency we observed is not caused by a reduced perisomatic synapse number, but rather by a reduced release probability.

Next, to test whether the GABAergic release probability is reduced, we measured the paired-pulse ratio (PPR) for GABAergic input onto pyramidal neurons (Fig. 4d, e, Supp. Table S2). PPR for input from PV^+^ neurons has been shown to be depressing, i.e. the response to the second stimulus is lower than the response to the first stimulus (Chang et al. 2014). We did indeed find a rapidly depressing paired-pulse response in *Ehmt1*^+*/*+^ slices (Fig. 4d, e). However, we found the PPR to be less depressing in the *Ehmt1*^+*/*−^, specifically at 100 ms, 150 ms, and 200 ms inter-stimulus interval (Fig. 4d, e), indicating that GABAergic release is affected in *Ehmt1*^+*/*−^ mice.

Next, we wondered whether the less-depressing PPR in *Ehmt1*^+*/*−^ is specific to the synapses originating from PV^+^ neurons. To this end, we blocked GABA release specifically from PV^+^ neurons by blocking their dominant Ca^2+^ channel (P/Q channels) via bath application of ω-Agatoxin-IVA (400 nM, Zaitsev et al. 2007). After ω-Agatoxin-IVA application, the remaining IPSC amplitude was reduced by approx. 85%, and remaining IPSC decay time was slower (Supplementary Figure S6), indicating that ω-Agatoxin-IVA blocked fast inhibitory transmission from PV^+^ neurons, in agreement with previous reports in the rat prefrontal cortex (Zaitsev et al. 2007). More importantly, following ω-Agatoxin-IVA application, both *Ehmt1*^+*/*+^ and *Ehmt1*^+*/*−^ showed similar PPRs (Fig. 4f, Supplementary Table S2), indicating that the PPR phenotype in *Ehmt1*^+*/*−^ without ω-Agatoxin-IVA originates from PV^+^ neurons.

We further wondered whether the less-depressing PPR in *Ehmt1*^+*/*−^ was caused by a reduced release probability (*P*_R_), or by a change in the vesicle pool available for release (readily releasable pool). To this end, we measured PPRs with elevated *P*_R_ by elevating the extracellular calcium level to 4 mM Ca^2+^. We found similar PPRs in *Ehmt1*^+*/*+^ under baseline (2 mM Ca^2+^) and elevated Ca^2+^ (4 mM Ca^2+^) conditions (Fig. 4g, Supplementary Table S2). This might indicate a ceiling effect for GABAergic release in the wild-type condition (see for a review: Neher 2015). In contrast, we found that *Ehmt1*^+*/*−^ neurons showed significantly more paired-pulse depression at 4 mM Ca^2+^ than at 2 mM Ca^2+^, specifically at 150, 200, and 500 ms ISI (Fig. 4h, Supplementary Table S2), which implies that *Ehmt1*^+*/*−^ mice might have a lower baseline release probability.

In order to fully quantify the release probability and readily releasable pool, we exhausted the synaptic pools with a depletion protocol consisting of trains of 100 stimuli at 10 Hz at elevated Ca^2+^ levels (4 mM; Fig. 4i). In both genotypes, we observed a rapid rundown of GABAergic responses, eventually reaching a steady state. However, in *Ehmt1*^+*/*−^, the relative amplitudes during the rundown were higher than in *Ehmt1*^+*/*+^ (Fig. 4i). We hypothesized that this finding could be consistent with a reduced release probability, but not necessarily a smaller vesicle pool. Specifically, if a smaller portion of the pool would get released with each subsequent pulse, then reaching the steady-state level would take more stimulations.

We therefore estimated both the readily releasable pool and release probability from the 10 Hz stimulus trains. Consistent with our hypothesis, we found that the readily releasable pool was unchanged between *Ehmt1*^+*/*+^ and *Ehmt1*^+*/*−^ at an estimated 7 initial stimulus amplitude sizes (Fig. 4j, Supplementary Table S2). The release probability, in contrast, was indeed significantly lower in *Ehmt1*^+*/*−^ (Fig. 4k, Supplementary Table S2), confirming our hypothesis and pinpointing the impaired GABAergic transmission in *Ehmt1*^+*/*−^ cortex to PV^+^ presynapses.

To conclude, our electrophysiological experiments paint a consistent picture of a reduced GABA release probability in *Ehmt1*^+*/*−^ A1 layer 2/3 at P14, specific to putative PV^+^ synapses. This synaptic deficit matches the delay in PV^+^ neuron maturation observed in the same region and time (A1 layer 2/3 at P14; Fig. 1c, d) and indicates an impairment in both maturation and function of PV^+^ neurons in the early sensory cortices of *Ehmt1*^+*/*−^ mice.

## Discussion

Here we describe a region- and time point-specific influence of *Ehmt1* haploinsufficiency on the development and function of PV^+^ neurons. In all three sensory cortical areas, we found a reduction of PV^+^ developmental markers in layer 4 at P14 (see Supplementary Fig. S4 for an overview). In *Ehmt1*^+*/*−^ A1 and V1, those markers normalized to wild-type levels by P28, indicating a delayed PV^+^ maturation. In contrast, in the *Ehmt1*^+*/*−^ S1 layer 4, PV^+^ maturation markers remained reduced into adulthood (P56), which implies an area-specific permanent immature state for S1 in *Ehmt1*^+*/*−^ mice. Genetic labelling coupled with whole-brain immunostaining and clearing using the iDisco + protocol (Renier et al. 2014, 2016) showed no difference between the genotypes at P56 in any primary sensory cortex (Supplementary Fig. S2), including S1 layer 4. This indicates that in *Ehmt1*^+*/*−^ mice, the generation and migration of MGE-derived (future PV^+^ and SST^+^ neurons) is not impaired in the primary sensory cortices, which raises the interesting possibility that continuous downregulation of PV^+^ neurons identified with anti-PV immunostainings might be an activity-dependent downregulation due to reduced circuit activity in the barrel cortex.

The “integrative” layer 2/3 was more variable across regions and time. In the A1 layer 2/3, we observed a developmental delay in *Ehmt1*^+*/*−^, similar to A1 layer 4. However, S1 and V1 layer 2/3 showed no differences between genotypes at any age (Supplementary Fig. S4). We then investigated whether the immature PV^+^ phenotype at P14 is also reflected at the functional level. We found that GABAergic input to A1 layer 2/3 pyramidal neurons is reduced in *Ehmt1*^+*/*−^. This reduction is selectively caused by a reduced GABA-release probability at the PV^+^ presynapse in *Ehmt1*^+*/*−^ cortex.

Auditory cortex is the only sensory area we tested where PV^+^ maturation was impaired in both L2/3 and 4 at P14, but normalized later in development. In mice, P14 is the middle of the critical period for the auditory cortical tonotopic map (Barkat et al. 2011; Takesian et al. 2018). It therefore seems that in *Ehmt1*^+*/*−^ mice, the auditory critical period might be shifted past P14, which is consistent with earlier studies in which the appearance of an acoustic startle response was delayed in *Ehmt1*^+*/*−^ by approximately one day, from P13.5 in *Ehmt1*^+*/*+^ to P14.5 in *Ehmt1*^+*/*−^ (Balemans et al. 2014).

This poses the question whether an early change in PV^+^ developmental trajectory causes lasting network defects in *Ehmt1*^+*/*−^ sensory cortices, even once the expression of PV and PNNs have normalized to wild-type levels. Evidence from other mouse models with changed PV^+^ developmental trajectories indicates that this could be the case. In *Mecp2*^*−/y*^ mice, for example, PV^+^ neurons develop precociously before the onset of vision, resulting in impaired sensory perception in adulthood (Durand et al. 2012; Patrizi et al. 2019) and a premature closure of the critical period (Krishnan et al. 2015). An early sensory change affects sensory-cortex dependent learning in adult mice, recently shown in *Mecp2*^+*/*−^ mice with pup retrieval, a learned maternal behavior that is dependent on active remodeling of PV^+^ neurons (Krishnan et al. 2017). Specifically, *Mecp2*^+*/*−^ females showed impaired PV and PNN remodeling following surrogate maternal experience, and consequentially displayed impaired pup-gathering behavior (Krishnan et al. 2017; Lau et al. 2020a, b).

In the primary visual cortex, we observe an early (P14) PV^+^ maturation delay in *Ehmt1*^+*/*−^ layer 4, but not in layer 2/3. P14 is just after the timepoint of eye opening in WT mice, and this event is delayed in *Ehmt1*^+*/*−^ mice, to P16 (Balemans et al. 2014). As a result, the reduced PV^+^ interneuron count in layer 4 at P14 could be due to a delay in the arrival of sensory information in *Ehmt1*^+*/*−^ mice. Interestingly, a delayed onset of visual perception has been linked to a delayed closure of the auditory critical period in wild-type mice (Mowery et al. 2016), which poses an interesting possibility of cross-modal plasticity.

In the *Ehmt1*^+*/*−^ somatosensory cortex layer 4, we found that the density of PV^+^ interneurons remains low into adulthood. A possible explanation might be a blurred representation of the barrel field, as for example in *Fmr1*^*−/y*^ mice (Juczewski et al. 2016). As PV^+^ interneurons are located in the barrel walls, a blurred representation might imply a permanently reduced PV^+^ interneuron count as well (Selby et al. 2007). Alternatively, the reduced PV^+^ count might reflect an inactive state of the whisker system in general, similar to a state of sensory deprivation (McRae et al. 2007), which would be consistent with the fact that genetic labelling of PV^+^ neurons did not show a difference between genotypes (Supp. Fig. S2c). The somatosensory system is the only of the sensory systems tested here that requires active movement (i.e. sweeping of the whiskers), with precisely timed sensorimotor integration between S1, secondary somatosensory cortex (S2) and motor cortical areas (see for a review: Petersen 2007). This type of precise, long-range connectivity between areas may be impaired in *Ehmt1*^+*/*−^ mice, for example due to dysregulated expression of guidance cues such as clustered Protocadherins (Iacono et al. 2018). Defects may also occur further upstream, as *Ehmt1* is also expressed in the developing whisker pad (Kleefstra et al. 2005) and during the development of sensory subcortical circuitry (Ebbers et al. 2016). Functionally, a permanently reduced PV^+^ interneuron activity such as caused by early whisker trimming has been linked to deficits in sensory acuity, hypersensitivity to stimuli (Antoine et al. 2019) and social deficits (Wolfe et al. 2011). As a consequence, the deficits in social and sensory behavior in *Ehmt1*^+*/*−^ mice may be partially mediated by a reduced S1 L4 inhibitory function (Balemans et al. 2010, 2013, 2014).

The emergence of PV and PNNs was approximately equal in our measurements, which indicates that PV and PNN are expressed at approximately the same rate (Supplementary Fig. S3). When we plot the percentage of PV^+^ cells that also express PNNs (Supplementary Fig. S1), we see that the percentage of double-labelled PV^+^ neurons is generally higher in layer 4 than layer 2/3, and higher in the somatosensory cortex than in either visual or auditory. Interestingly, we only observe significant reductions in the percentage of double-labelled cells in *Ehmt1*^+*/*−^ during the critical period for the respective region, with A1 layer 2/3 and 4 at P14, S1 layer 2/3 at P14, and V layer 2/3 at P28 (Supplementary Figure S1). PNNs are honeycomb-like structures that enwrap soma and proximal dendrites of (mostly) PV^+^ neurons (review: Testa et al. 2019). Recent reports indicate the activity level of PV^+^ neurons cell-autonomously regulate the production of PNNs and PNN-modifying enzymes even in adulthood (Devienne et al. 2019). In the *Fmr1*^*−/y*^ mouse, a delay of both PV^+^ and PNN development is visible, which in this mouse is caused by an overproduction of the PNN modifier MMP-9 during the auditory critical period (Wen et al. 2018). Interestingly, while the PV^+^ developmental trajectory in *Fmr1*^*−/y*^ matches our *Ehmt1*^+*/*−^ findings, MMP-9 is downregulated during development in the *Ehmt1*^+*/*−^ cortex (Iacono et al. 2017). Thus, here we observe a phenotypic convergence despite molecular divergence in the PNN modification pathway.

PNNs enhance the capacity of PV^+^ neurons for high-frequency firing by reducing the membrane capacitance (Tewari et al. 2018) and clustering ion channels (Favuzzi et al. 2017). PNNs also bind extracellular molecules that are important for the developmental trajectory of PV^+^ neurons, for example the axon guidance cues Semaphorin 3a/b (De Winter et al. 2016) and the trans-synaptic transcription factor Otx2. This homeoprotein is not produced anywhere in the cortex, but rather in sensory afferents and the choroid plexus (Spatazza et al. 2013; Sugiyama et al. 2008). Otx2 binds to PNNs and enters the future PV^+^ cell, where it triggers a genetic program for critical period plasticity (Beurdeley et al. 2012; Prochiantz and Di Nardo 2015; Testa et al. 2019) by upregulation of *Gadd45b*, a DNA demethylase that orchestrates the large-scale change in the transcriptomic landscape during the critical period, including clustering of MeCP2 in the nucleus (Apulei et al. 2019), where it has been shown to directly bind to and methylate the *Pvalb* promoter region (Patrizi et al. 2019). Interestingly, *MeCP2* has recently been shown to be a direct target of *Ehmt1* in non-neuronal cells (Choi et al. 2018). Besides signalling by Otx2, another interesting candidate might be the signalling molecule BDNF, which is both important for PV^+^ neuron maturation (Berghuis et al. 2006; Huang et al. 1999) and negatively regulated by EHMT1 (Benevento et al. 2016). Specifically, EHMT1 methylates histones around the activity-dependent *Bdnf* promoter IV in response to network silencing, leading to a failure of activity-dependent *Bdnf* repression in *Ehmt1*^+*/*−^ neurons (Benevento et al. 2016). However, it is currently unknown whether EHMT1 also plays a role in *Bdnf* upregulation via promoters other than *Bdnf* *IV*, as happens during normal cortical development (Du et al. 2018). Interestingly, emerging evidence links BDNF signaling to the *MeCP2*^*−/y*^ synaptic phenotype as well (Sampathkumar et al. 2016), even though also in this case a precise molecular mechanism still remains to be found. Quite possibly, several different signaling systems (e.g. Otx2 and BDNF, as well as circuit activity levels) could converge on PV^+^ neurons during the critical period, triggering a common genetic program including PV expression and PNN production. In turn, the actual execution of this of this genetic program would require precisely targeted epigenetic modulation, possibly including Gadd45b and MeCP2 as well as EHMT1. Consequentially, an *Ehmt1* haploinsufficiency could cause an incomplete transition from the pre-critical period transcriptomic state to the post-critical period transcriptomic state, thus delaying the critical period.

A series of recent reports indicates that reduced feedforward inhibition is a common feature in several mouse models of ASD (Antoine et al. 2019; Banerjee et al. 2016). Across at least five different mouse models of ASD, feedforward inhibition was found to be much more impaired than feedforward excitation, resulting in a shifted *E*/*I* balance towards excitation. Our results are consistent with this interpretation, as we find a decreased GABAergic transmission (Fig. 4). In *Ehmt1-*deficient cells from rodents and humans, excitatory transmission is not impaired at resting membrane potential, but summation of excitatory input is impacted by an upregulation of NMDA receptors (Frega et al. 2019, 2020). Antoine et al. (2019) argue that excitation and inhibition are matched to yield stable in vivo firing patterns. This homeostatic regulation might still prove maladaptive in some situations, resulting in widened integration time windows and an inability to compensate for extremely high or low input levels. Also here, *Ehmt1*^+*/*−^ mice fit the template of impaired adaptation, as excitatory synaptic scaling-up in response to input deprivation is impaired in *Ehmt1*^+*/*−^ mice (Benevento et al. 2016). To summarize, in *Ehmt1*^+*/*−^ mice we find a region- and layer-specific developmental delay of PV^+^ neuron maturation in the three main sensory cortical areas. PV^+^ neurons also are functionally immature, showing a reduced inhibitory output specifically caused by a reduced release probability. As a consequence, we predict a delayed closing of the critical period in auditory and somatosensory cortex. Both the onset and the closing of the critical period require coordinated changes in gene expression in PV^+^ neurons (Apulei et al. 2019), which has been shown to require epigenetic remodeling such as by the DNA methylation reader MeCP2 (Krishnan et al. 2015), which interestingly was recently shown to be a direct target of *Ehmt1* in non-neuronal cells (Choi et al. 2018). It is therefore conceivable that a loss of *Ehmt1* would lead to a cell-autonomous failure of epigenetic remodeling, resulting in an incomplete repression of previously expressed gene sets and thus a slowed transition into and out of the CP. This concept could be generalized to other changes in gene expression, and indeed is consistent with activity-dependent scaling of excitatory synapses in *Ehmt1*^+*/*−^ neurons (Benevento et al. 2016) and activity-dependent rapid methylation of early-response genes by EHMT1 and EHMT2 (Gupta-Agarwal et al. 2012). We predict that finding out how the epigenetic landscape changes in inhibitory neurons—and when those changes fail—will be an important addition to solving the secret of the dynamic brain.

## Electronic supplementary material

Below is the link to the electronic supplementary material.

Supplementary file1 (DOCX 2239 kb)

Supplementary file2 (XLSX 31 kb)

Supplementary file3 (XLSX 22 kb)
