# Supplementary material for: EHMT1 regulates Parvalbumin-positive interneuron development and GABAergic input in sensory cortical areas
Source: Brain Struct Funct. 2020 Sep 25;225(9):2701–16. doi: 10.1007/s00429-020-02149-9 (PMC7674571; doi:10.1007/s00429-020-02149-9)
Supplement: Supplementary file 1 — Supplementary file1 (DOCX 2239 kb) [file 429_2020_2149_MOESM1_ESM.docx]

**Supplementary Data**

***Ehmt1* regulates Parvalbumin-positive interneuron development and GABAergic input in sensory cortical areas**

Moritz Negwer^1^, Karol Piera^1^, Rick Hesen^1^, Lukas Lütje^1^, Lynn Aarts^1^, Dirk Schubert^2^, and Nael Nadif Kasri^1, 2,*^

^1^Department of Human Genetics, Radboudumc, Donders Institute for Brain, Cognition, and Behaviour, 6500 HB Nijmegen, the Netherlands

^2^Department of Cognitive Neuroscience, Radboudumc, Donders Institute for Brain, Cognition and Behaviour, 6500 HB Nijmegen, the Netherlands

* To whom correspondence should be addressed: [n.nadif@donders.ru.nl](mailto:n.nadif@donders.ru.nl)

**Supplementary Figures**


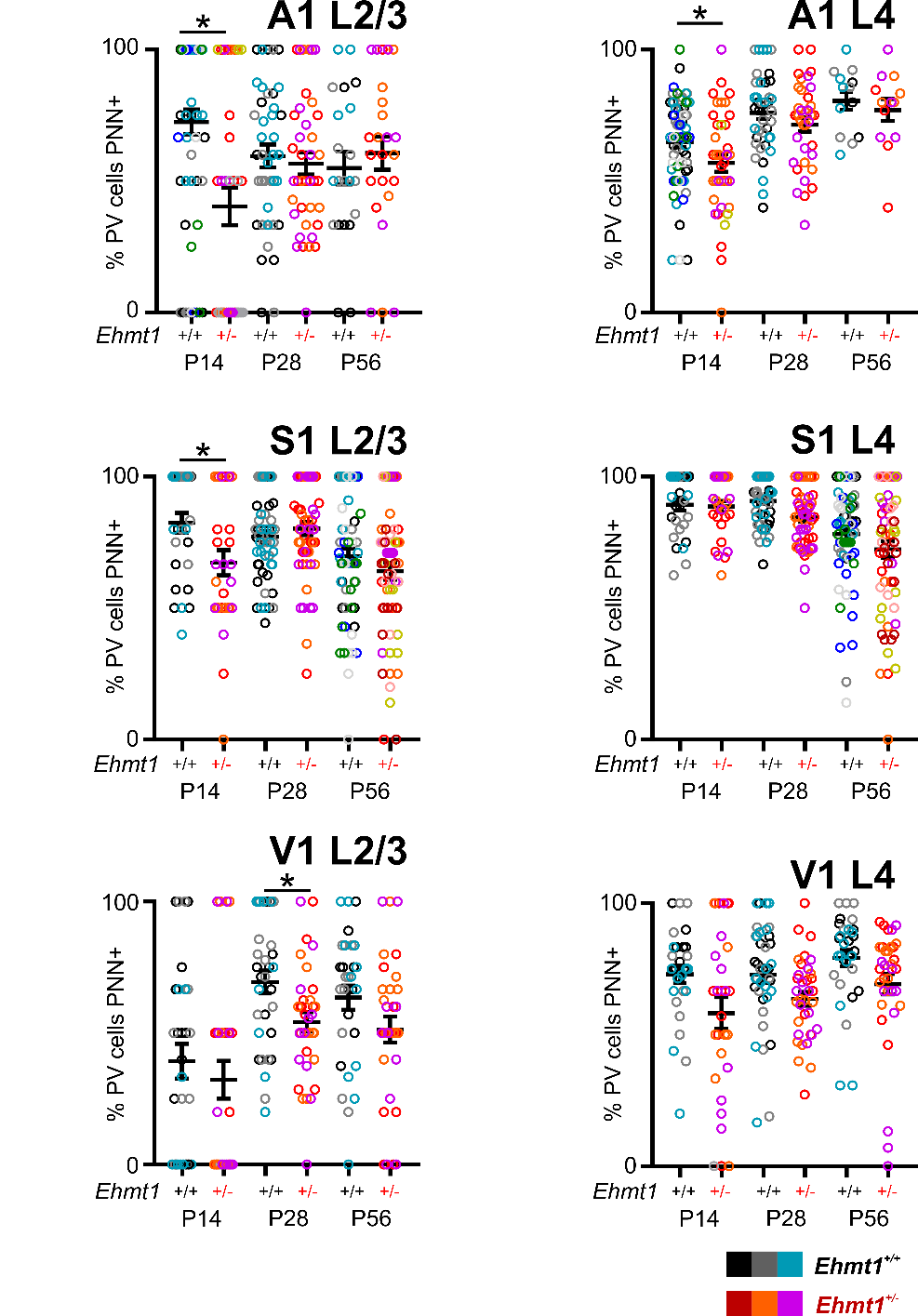


**Supplementary Figure S1, related to Figs 1-3:** Percentages of PV^+^ cells that are double-labelled with PNNs, per age and region. Each dot represents one image, color-code per mouse (in the same age cohort). Bars are mean ± SEM. * p<0.05, nested ANOVA.

**
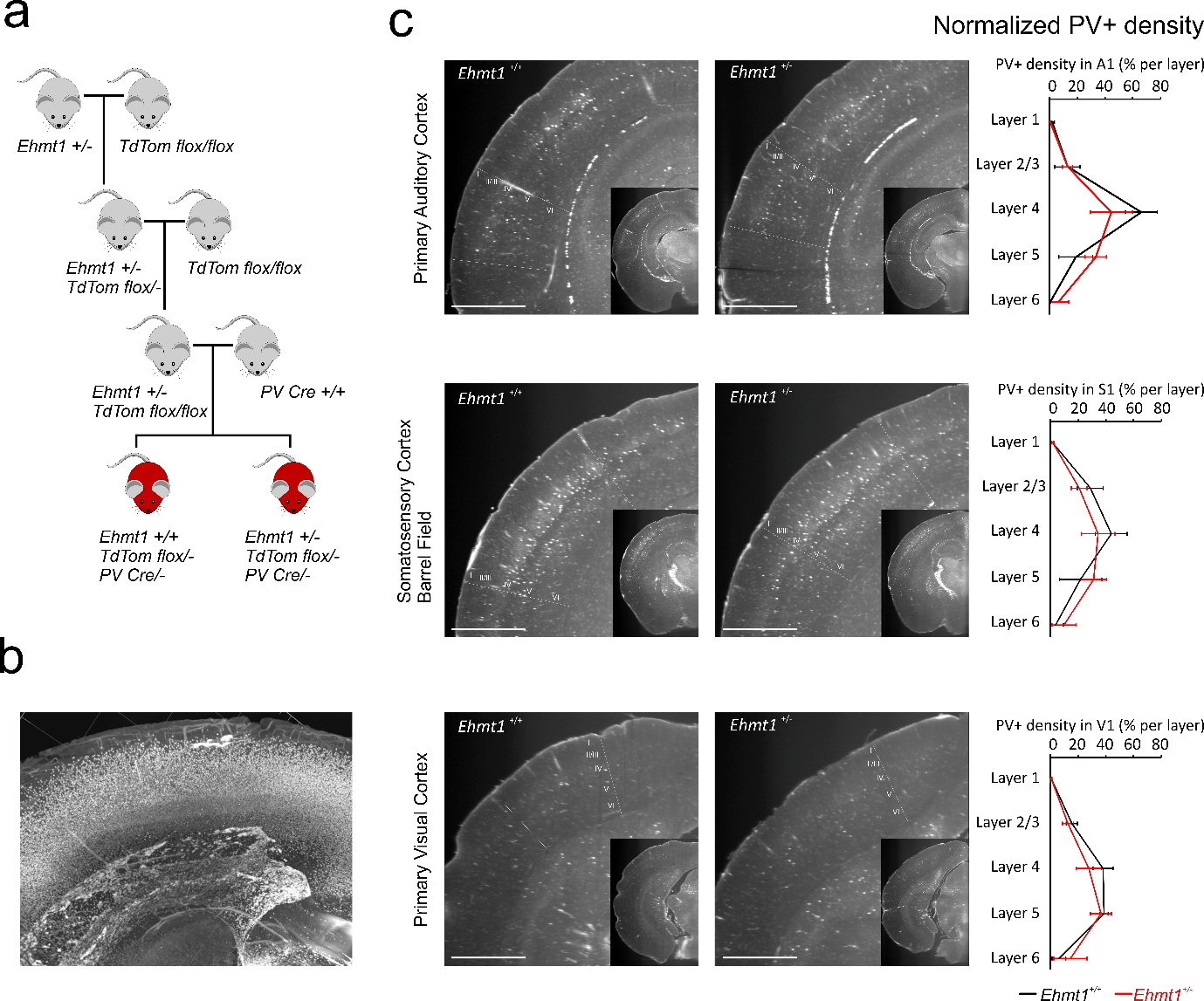
**

**Supplementary Figure S2, related to Figs 1-3:** Genetic labelling of PV-expressing neurons and visualization with whole-brain immunolabelling and clearing with the iDISCO+ technique. **a** Breeding scheme to generate mice with labelled PV-expressing neurons. Litter-matched mice from the last breeding step (marked in red) were used for the experiments at P56, each expressing PV ^Cre/-^;Tdtom ^flox/-^,and were either *Ehmt1^+/+^* or *Ehmt1^+/-^*. **b** Rendering of Tdtomato-labelled cells, virtual 600µm slice of an entire hemisphere, coronal orientation. **c Left**, single coronal planes (1 px, 2.95 µm thickness) of auditory, somatosensory, and visual cortex. Scale bar: 1 mm. Inset: zoomed out image of the image plane across the entire hemisphere. **Right**: Quantification of TdTomato-expressing neurons across layers in primary auditory, somatosensory, and visual cortices. No difference was found in either region (n= 3/5 *Ehmt1^+/+^*/*Ehmt1^+/-^* brains at P56, Student’s *t* test).


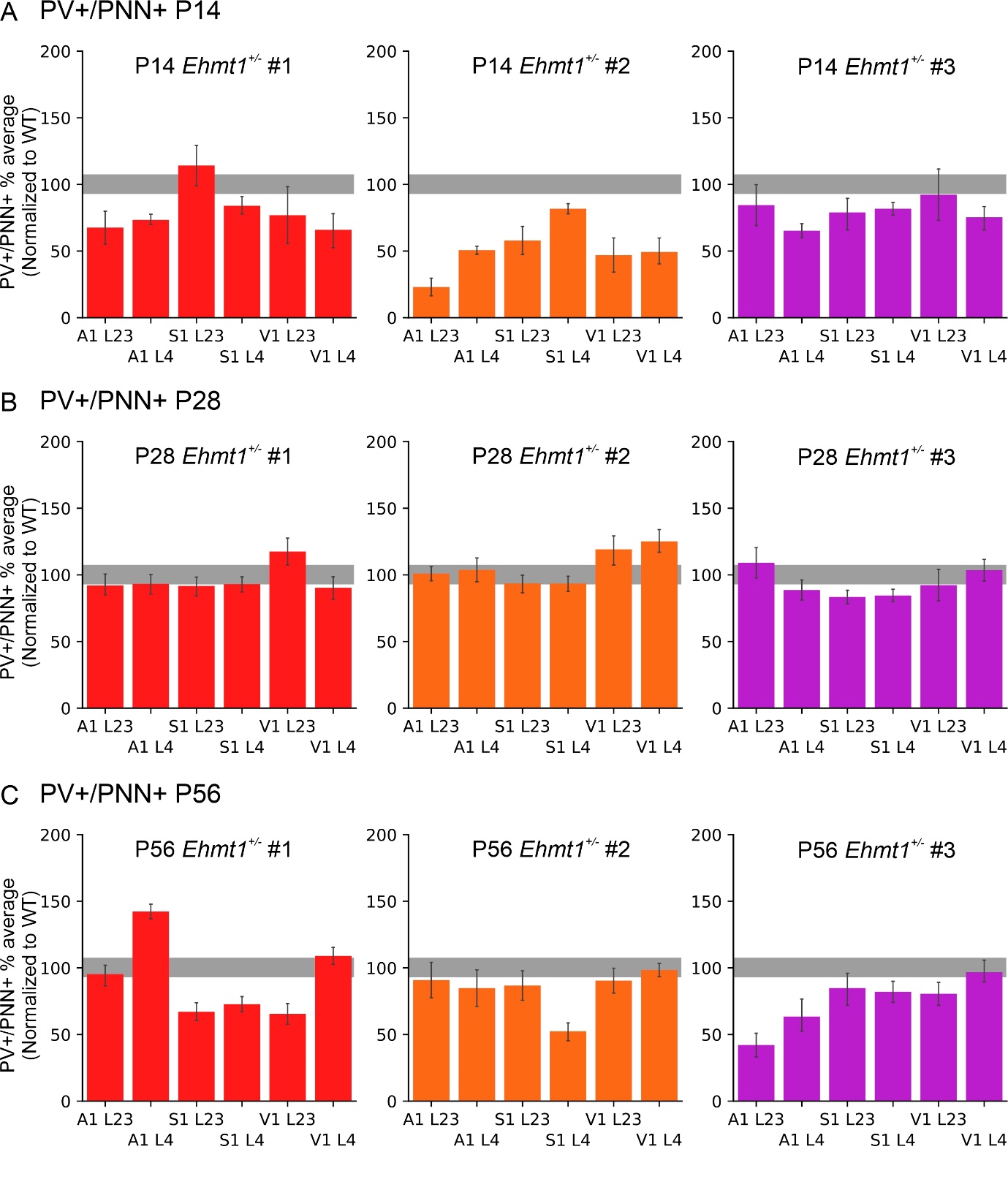


**Supplementary Figure S3, related to Figs. 1-3:** PV^+^/PNN^+^ density of *Ehmt1^+/-^*, normalized to the age-matched *Ehmt1^+/+^* littermates. Plotted here are the *Ehmt1^+/-^* data per mouse, for the mice where we have data from all sensory areas, color-coded the same as in Figs 1-3. **a**, normalized PV^+^/PNN^+^ density at P14; **b**, normalized PV^+^/PNN^+^ density at P28; **c**, normalized PV^+^/PNN^+^ density at P56. The grey bar represents the mean ± SEM of the *Ehmt1^+/+^* littermates (the mean is per definition 100% in this normalization). N=3/3 *Ehmt1^+/+^* / *Ehmt1^+/-^* per age. Please see Supplementary Table 1 for details on the numbers plotted here.


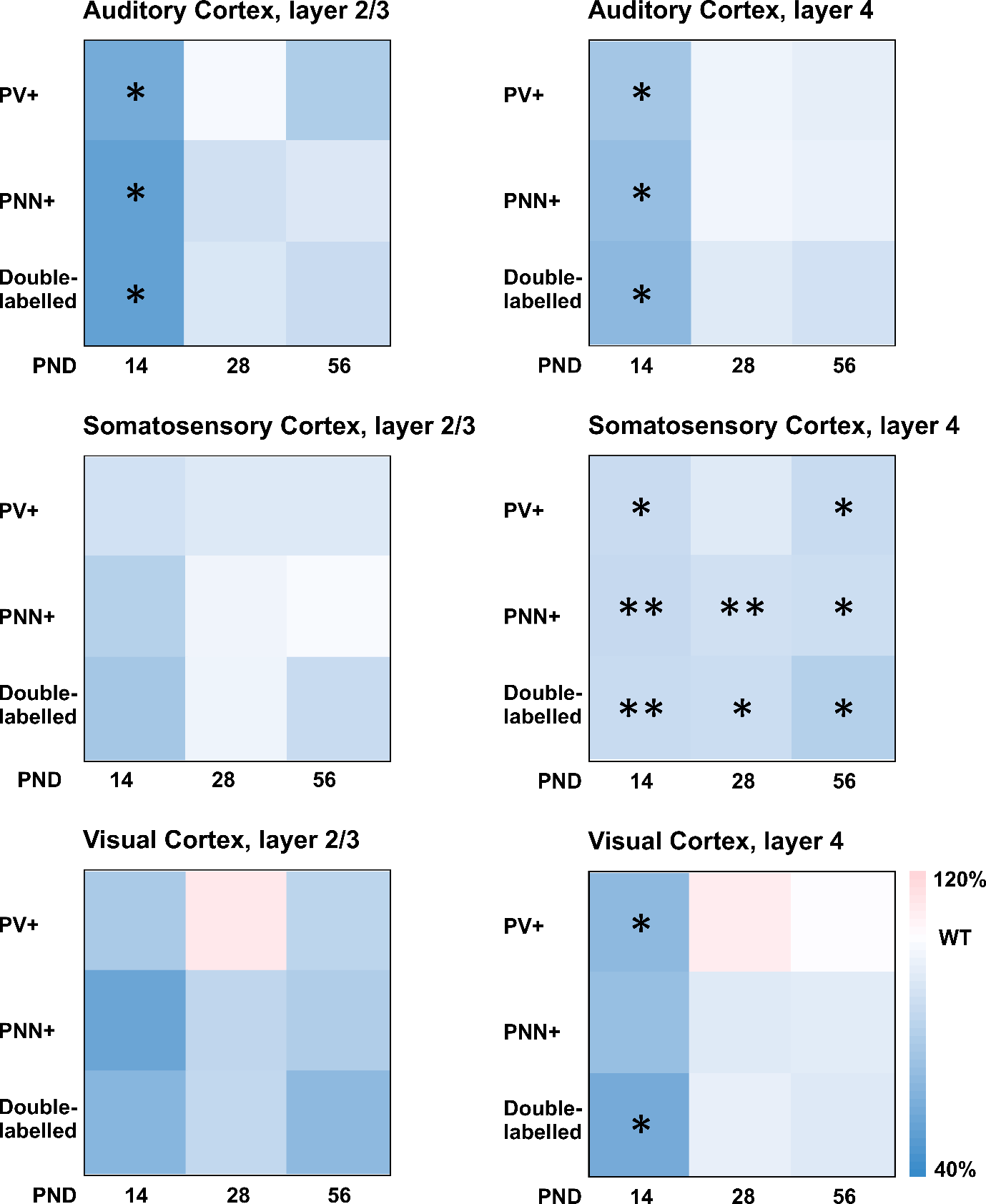


**Supplementary Figure S4, related to Figures 1-3:**

Graphical summary of Figures 1-3. Per graphic: X-axis = age group (P14, P28, P56), Y-Axis = PV^+^ density, PNN^+^ density, PV/PNN double-labelled density (each in cells /mm²). The color indicates change in *Ehmt1^+/-^* w.r.t. wild-type in the same age and region. White = no change, blue = reduced in *Ehmt1^+/-^* compared to *Ehmt1^+/+^*, red = increased in *Ehmt1^+/-^* compared to *Ehmt1^+/+^*. * p<0.05, ** p<0.01, nested ANOVA


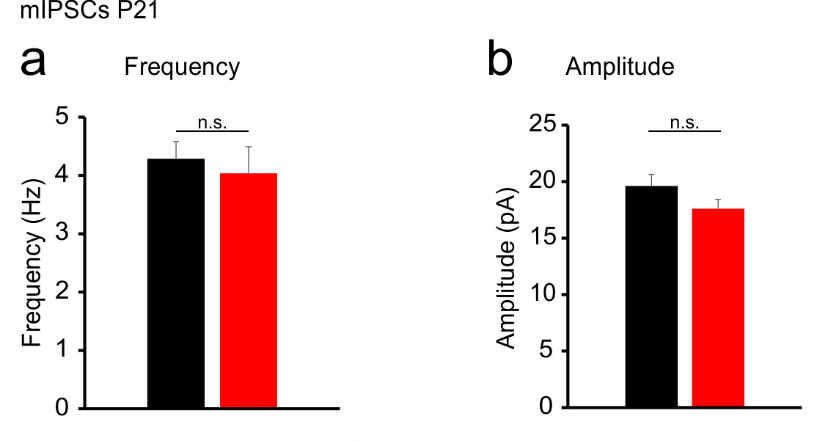


**Supplementary Figure S5, related to Figure 4b:**

**a-b:** Miniature inhibitory postsynaptic potentials (mIPSCs) in pyramidal neurons in auditory cortex layer 2/3, measured at P21 (one week after the measurements in Fig. 4). We found no significant differences in either mIPSC Frequency (a), nor Amplitude (b). Both: n.s., Bonferroni-corrected *t*-test. N=14/15 *Ehmt1^+/+^/ Ehmt1^+/-^* cells.

**
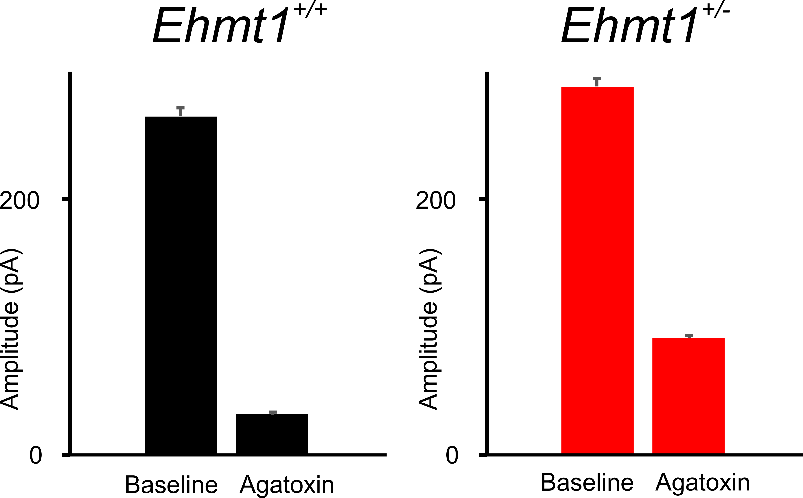
**

**Supplementary Figure S6, related to Figure 4f:**

Effects of ω-Agatoxin-IVA application on IPSC amplitude. Amplitude from evoked IPSCs pre- and post-Agatoxin application, measured in the same cell. Averages from 2/2 *Ehmt1^+/+^/ Ehmt1^+/-^* cells, 20 stimuli with 30s ISI per condition. Following ω-Agatoxin-IVA application, the amplitude drops by approx. 85% on average.
